# Obese Older Type 2 Diabetes Mellitus Patients with Muscle Insulin Resistance Benefit from an Enriched Protein Drink during Combined Lifestyle Intervention: The PROBE Study

**DOI:** 10.3390/nu12102979

**Published:** 2020-09-29

**Authors:** Wilrike J. Pasman, Robert G. Memelink, Johan de Vogel-Van den Bosch, Mark P. V. Begieneman, Willem J. van den Brink, Peter J. M. Weijs, Suzan Wopereis

**Affiliations:** 1 Netherlands Organisation for Applied Scientific Research (TNO), 3704 HE Zeist, The Netherlands; mpvbegieneman@protonmail.com (M.P.V.B.); willem.vandenbrink@tno.nl (W.J.v.d.B.); suzan.wopereis@tno.nl (S.W.); 2 Department of Nutrition and Dietetics, Faculty of Sports and Nutrition, Center of Expertise Urban Vitality, Amsterdam University of Applied Sciences, 1067 SM Amsterdam, The Netherlands; r.g.memelink@hva.nl (R.G.M.); p.j.m.weijs@hva.nl (P.J.M.W.); 3 Danone Nutricia Research, 3584 CT Utrecht, The Netherlands; johan.devogel@nutricia.com; 4 Department of Nutrition and Dietetics, Amsterdam University Medical Centres, Vrije Universiteit, 1081 HV Amsterdam, The Netherlands

**Keywords:** combined lifestyle intervention, diabetic subtypes, muscle insulin resistance, oral glucose tolerance test (OGTT), weight loss

## Abstract

(1) Background: Recent research showed that subtypes of patients with type 2 diabetes may differ in response to lifestyle interventions based on their organ-specific insulin resistance (IR). (2) Methods: 123 Subjects with type 2 diabetes were randomized into 13-week lifestyle intervention, receiving either an enriched protein drink (protein+) or an isocaloric control drink (control). Before and after the intervention, anthropometrical and physiological data was collected. An oral glucose tolerance test was used to calculate indices representing organ insulin resistance (muscle, liver, and adipose tissue) and β-cell functioning. In 82 study-compliant subjects (per-protocol), we retrospectively examined the intervention effect in patients with muscle IR (MIR, *n* = 42) and without MIR (no-MIR, *n* = 40). (3) Results: Only in patients from the MIR subgroup that received protein^+^ drink, fasting plasma glucose and insulin, whole body, liver and adipose IR, and appendicular skeletal muscle mass improved versus control. Lifestyle intervention improved body weight and fat mass in both subgroups. Furthermore, for the MIR subgroup decreased systolic blood pressure and increased VO_2_peak and for the no-MIR subgroup, a decreased 2-h glucose concentration was found. (4) Conclusions: Enriched protein drink during combined lifestyle intervention seems to be especially effective on increasing muscle mass and improving insulin resistance in obese older, type 2 diabetes patients with muscle IR.

## 1. Introduction

A key therapeutic goal in the treatment of obese type 2 diabetes (mellitus) patients is weight loss. Several studies using energy restriction and weight loss interventions showed beneficial effects on the metabolic state, including reduced HbA1c and lipoprotein levels, reduced hepatic and pancreatic fat content, improved β-cell function (BCF) and hepatic insulin sensitivity, and even remission of type 2 diabetes [1,2,3,4]. However, a disadvantageous effect of weight loss interventions is the loss of muscle mass that can account for about 30% of total weight loss [5]. Indeed, in a 1-year controlled, randomized trial that evaluated the effects of diet and exercise on weight loss in obese older adults, 39% and 22% of the weight loss found after 6 months originated from lean mass in the diet group and diet-exercise group [6] respectively. In addition, aging itself is also known to be associated with muscle mass loss [7]. This is especially problematic in the obese older diabetic population, as these individuals not only suffer from decreased glycemic control but also are at risk of losing skeletal muscle mass [8]. This loss of muscle mass is most undesirable, because skeletal muscle primarily accounts for about 80% of insulin-mediated glucose uptake [9]. Skeletal muscle is also an important metabolic organ, oxidizing sugars and fats to produce ATP to enable muscle activity. A vicious circle may arise if the weight loss-induced muscle mass loss, in addition to the age-induced muscle mass loss, further worsens glucose uptake and glycemic control [10]. This stresses the importance of weight loss interventions that preserve muscle mass for obese, older type 2 diabetes patients.

Both resistance exercise and high-protein intake have been shown to help maintain and protect muscle mass during weight loss [11,12,13]. Additionally, resistance exercise training also increases muscle insulin sensitivity in older type 2 diabetes adults [10]. In the recent PROBE study, improved muscle mass and glycemic control in obese, older type 2 diabetes patients was achieved using a whey protein drink enriched with leucine and vitamin D during a combined lifestyle intervention consisting of resistance exercise and energy restriction [14]. 

However, dietary interventions often lack consistent effects that may be related to differences in genotype, lifestyle habits, and environmental factors. Due to this individual variability, people may react differently to metabolic stressors and interventions, so-called ‘metabolic or phenotypic flexibility’ [15]. Also, several different organs and processes are involved in metabolic homeostasis and can contribute to disruptions in these processes [16]. The degree of β-cell function and insulin resistance (IR) of the primary involved organs (liver and skeletal muscle) in type 2 diabetes, may therefore greatly affect the individual metabolic flexibility. Recently, it has been shown that based on insulin resistance of muscle, liver, or a combination thereof, type 2 diabetes subtypes could be identified that differed in their long-term health response after two different dietary interventions [17]. Thus, identifying the individual diabetic phenotype may be important in determining the effectiveness of interventions. Diabetic subtypes can be discriminated using formulas with fasting plasma glucose and insulin in combination with plasma values of a 5-points-2 h oral glucose tolerance test (OGTT). These calculations describe different phenotypes based on the level of beta cell activity, whole body insulin sensitivity, and muscle or liver insulin resistance [18,19,20]. 

To study whether obese, older type 2 diabetes subtypes have a different response to a lifestyle intervention with or without a protein drink, as a secondary objective of the main study, we retrospectively analyzed diabetes subgroup effects from patients participating in the PROBE study. Appendicular muscle mass was found to be increased during the lifestyle intervention when a protein drink was used [14], we therefore focused on muscle mass and insulin resistance of muscle. We hypothesized that especially patients with muscle insulin resistance have impaired muscle function and may benefit from muscle stimulating interventions like exercise training and protein intake. Muscle insulin resistance could be an important variable to identify subgroups that may benefit more or less from the lifestyle and protein drink intervention, therefore the data were compared for diabetic patients with muscle insulin resistance (MIR) and without MIR (no-MIR) subgroups based on the muscle insulin sensitivity index (MISI) [18]. With this index, the change in blood glucose is shown over time relative to insulin, meaning that insulin sensitive people will have a relatively high uptake of glucose by the muscle over time with a certain concentration of insulin. Muscle of patients that are insulin resistant will respond less well to insulin and, as a consequence, remove less glucose from the blood over time, resulting in a low MISI value. This subgrouping of patients may provide further insight and suggestions for mechanisms of importance for glycemic control and may be used to treat type 2 diabetes patients more efficiently.

## 2. Materials and Methods

### 2.1. Subjects

This study was conducted as described elsewhere [14]. In short, 123 subjects were recruited and eligible for study participation if they were aged 55 years or older, had type 2 diabetes (defined by use of medication for type 2 diabetes) or pre-diabetes (defined as a blood hemoglobin A1c level (HbA1c) ≥ 43 mmol/mL (6.1%)) and had either a body mass index (BMI) of >30.0 kg/m^2^ or a BMI > 27.0 kg/m^2^ in combination with a waist circumference of >88 cm for women and >102 cm for men. Exclusion criteria included cardiac infarcts and/or cardiac surgery within three months prior to baseline; any malignant disease in the last five years or renal, hepatic or gastrointestinal diseases; specific medication or diets and alcohol or drug abuse. The study was approved by the Medical Ethics Committee (NL46790.056.14) Assen June 2014, The Netherlands, in accordance with the Helsinki Declaration of 1975 as revised in Brazil, 2013. The study has been registered at www.trialregister.nl, NTR4497. Written informed consent was obtained from all subjects. The study was conducted from September 2014 till January 2017. 

### 2.2. Study Design

The study was a randomized, controlled, double blind 13-wk trial [14]. Subjects were randomized into either the test group or control group and followed a hypocaloric diet of 600 kcal below estimated energy needs (based on measured resting energy expenditure and estimated physical activity level using an accelerometer). Subjects received six individual dietary counseling sessions with a dietitian and six group sessions on alternating weeks together under supervision of a dietitian to facilitate a 600 kcal energy deficit with their food intake. The exercise program, consisted of three 1-h group training sessions per week together at a fitness center, and included resistance exercise and high intensity interval training (HIIT). The training sessions were supervised by a sports instructor. During the combined lifestyle intervention, the test group received a study drink 10 times a week: one serving daily with breakfast and one extra after each training session. Data of subjects was obtained at baseline and after 13 weeks of intervention. 

### 2.3. Study Products

In the study, drinks were provided by Danone Nutricia Research, Utrecht, The Netherlands. The test drink was a whey protein drink enriched with leucine and vitamin D, containing 21 g leucine-enriched whey protein (3 g total leucine), 9 g carbohydrates, 3 g fat, 800 IU cholecalciferol (vitamin D3), and a mixture of vitamins, minerals, and fibers, referred to as protein^+^ (FortiFit^®^). The control product contained 25 g carbohydrates and 6 g fat. Both products were similar in taste and appearance, isocaloric (150 kcal per serving), and were dissolved in 150 mL water just before consumption. The supplement was consumed daily and after each training session, so a total of ten consumptions per week. On average the protein intake for the protein^+^ users therefore increased with 28 g a day, or 112 kCal, representing about 6% of the daily energy intake. A detailed description of the study products is provided in Appendix A. 

### 2.4. Clinical Chemistry and Glycemic Control

To standardize the OGTTs subjects were instructed before the OGTTs: not to participate in any vigorous exercise 48 h; to refrain from alcohol for 24 h; have a standardized dinner the evening; not to eat, drink and smoke for 12 h; drinking some water was allowed till two hours before the OGTT. Upon overnight fasting an OGTT was performed at baseline and after 13 weeks of intervention. Venous blood samples were taken prior to intake (t = 0), and at 30, 60, 90 and 120 min after intake of a 300 mL 75 g glucose solution (Added Pharma). Plasma heparin samples were used for measurements of glucose (mmol/L) and insulin (pmol/L). Plasma samples taken prior to OGTT were also assessed for HbAc1 (mmol/mol) and plasma lipids (FFA, triglycerides, High-Density Lipoprotein, Low-Density Lipoprotein, and total cholesterol) and serum calcidiol (nmol/L) was assessed according to standard procedures in the laboratory of the Vrije Universiteit Medical Centre, Amsterdam, The Netherlands. Leucine analysis was based on the AccQTag method of Waters measured with HP-LC from a fasting dry blood spot sample at TNO, Zeist, The Netherlands.

### 2.5. Subtyping of Type 2 Diabetes

To define if a subject had muscle IR as the diabetic subtype, the muscle insulin sensitivity index (MISI) [18] was calculated from the OGTT. Additionally, the Matsuda index [19], hepatic insulin resistance index (HIRI) [18], disposition index (DI) [20], and the adipose tissue insulin resistance index (Adipo-IR) [21] were also calculated as measures for respectively systemic insulin sensitivity, hepatic insulin resistance, β-cell function (BCF), and adipose tissue insulin resistance (Table 1). Units used for calculations of the indices were in mg/dL for glucose and U/L for insulin. Using the data of DiOGenes [22], CORDIOPREV [23], and PhenFlex [24,25] studies, cut-off values for MISI were determined. These values were calculated and validated by using data of more than 1000 subjects with different health status: healthy subjects, subjects with prediabetes, and patients with undiagnosed and clinically diagnosed type 2 diabetes. The cut-off used for MISI was −1; subjects with values ≤ −1 had no MIR, subjects with values > −1 had MIR. In the present analysis, we calculated the MISI of all subjects at baseline and categorized them based on their MISI value in the MIR or no-MIR group, respectively.

### 2.6. Physical Measurements

Body weight was measured using a weighing scale (Life Measurement, Inc., Concord, CA, USA), body height using a wall mounted stadiometer (De Grood DGI 250D; De Grood metaaltechniek, Nijmegen, The Netherlands), and waist circumference using measuring tape. Body weight and height were used to calculate the body mass index (BMI). Appendicular skeletal muscle mass (ASMM) (kg), fat mass (kg), and visceral adipose tissue (VAT; cm^2^) were measured by dual-energy X-ray absorptiometry (DXA; Discovery A, Hologic), using a whole body scan. 

Three exercise tests were conducted to measure physical performance. The 10-RM leg press strength (kg) was measured using a leg press machine (Technogym Selection; Technogym, The Netherlands) Knee extension power (Watt) was measured using a leg extension machine (Technogym Selection; Technogym (Humac 360; CSMi) Technogym, The Netherlands) [26]. VO_2_peak (L/min) was measured using a steep ramp test performed on a cycle ergometer (Quark RMR/CPET; Cosmed, Rome, Italy) [27].

### 2.7. Statistics

The number of subjects in the randomized clinical trial (RCT) was based on the study of Verreijen et al. [13] evaluating the effect of the same test drink during intentional weight loss in older adults with obesity. A sample size of 44 per arm provided 80% power to detect an absolute difference of 0.92 kg leg muscle mass with a standard deviations (SD) of 1.51 kg and *p* < 0.05 (2-sided). Assuming a dropout rate of 25%, 118 subjects were needed for the study. Since subjects were enrolled in 5 different clusters, we aimed to enroll approximately 24 subjects per cluster. Finally, 123 subjects were enrolled in the study [14].

The data from the PROBE study were analyzed retrospectively, comparing the effect of test versus control in the MIR subgroup and no-MIR subgroup separately. To ensure high quality data, of the 123 subjects only those subjects were selected who consumed at least 7 out of 10 weekly study drinks (test or control), attended at least 2 out of 3 weekly training sessions (94 subjects), and completed the OGTT at start and end of the study (82 subjects) (per protocol analysis). 

Observations having a residual >3 times the root mean square error of the model were removed as statistical outlier. Before statistical analysis, variables visually showing increased residual variation with higher fitted values were transformed by taking their natural logarithm, while the MISI was logarithmically transformed after substituting the ‘0’ values by −0.1 and multiplying all values by −1 for proper statistical evaluation (due to non-linear distribution of the data log-transformation was needed). 

Mixed linear models were used to analyze each dependent outcome variable with ‘treatment’ (test vs. control) and ‘time’ (0 vs. 13 weeks) and their interaction as fixed independent effects, and ‘subject’ as random independent factor. This was performed for the MIR and the no-MIR subgroup separately, since baseline characteristics of the two subgroups were different for their diabetic status (Table 2) not allowing for a direct comparison between subgroups. No other subgroups were examined because the numbers of patients was unbalanced and too low. Interaction effects of time x treatment with *p* < 0.05 were further evaluated in a post hoc analysis to investigate whether a ‘time’ effect occurred in the test, the control group, or both. A post-hoc (two-sided student t-test after Bonferroni multiple comparison correction) effect with *p* < 0.05 was considered significant. Mean and SD were calculated for all outcomes. Statistical analyses were performed using R version 3.6.0 (www.r-project.org). 

### 2.8. Data and Resource Availability

The data sets generated during and/or analysed during the current study are available from the corresponding author upon reasonable request.

## 3. Results

### 3.1. Study Population

Eighty-two out of 123 subjects, who were compliant with respect to the PROBE study protocol and had complete OGTT data at baseline (week 0) and at the end of the intervention (week 13), were used for the retrospective type 2 diabetes subgroup analysis. The descriptive characteristics as presented in Table 2 were similar for the 82 subjects included as for the 41 excluded subjects of this retrospective analysis (data not shown). The subgroups MIR and no-MIR had similar baseline characteristics (see Table 2). 

Based on the MISI, 42 subjects were found to have muscle IR (MIR) against 40 subjects that did not (no-MIR). Table 2 shows some descriptive characteristics of the MIR and no-MIR subgroups, specified by protein^+^ and control group. The protein^+^ and control groups were comparable in terms of baseline characteristics within MIR as well as in no-MIR (NS). Although equal numbers of participants were found for MIR (*n* = 42) and no-MIR (*n* = 40), some main characteristics were different between the two subgroups. The MIR group has more males (75 vs. 55%), more prediabetics (26 vs. 5%) and related to that a lower diabetes duration (average median of 45 months vs. average median of 100 months) as compared to the no-MIR subgroup.

### 3.2. Protein^+^ Drink Effect within the Muscle Insulin Resistance Subgroup

Table 3 presents the intervention effect of the MIR subgroup. A significantly greater change over time was seen for the protein^+^ drink than for the control drink on Calcidiol (*p* < 0.001), fasting plasma glucose (FPG) (*p* = 0.020), fasting plasma insulin (FPI) (*p* = 0.029), Matsuda index (*p* = 0.002), HIRI (*p* = 0.019), Adipo-IR (*p* = 0.043), and ASMM (*p* = 0.043). Post-hoc analysis revealed that these effects were all explained by a significant improvement in the protein^+^ group alone: Calcidiol (+17.5 nmol/L, *p* < 0.001), FPG (−0.7 mmol/L, *p* = 0.002), FPI (−21.2 pmol/L, *p* = 0.031), HIRI (−700, *p* = 0.004) and Adipo-IR (−23.8, *p* = 0.011) all decreased, while Matsuda index (+0.47, *p* < 0.001) and ASMM (+0.54 kg, *p* = 0.041) increased after 13 weeks of intervention. 

### 3.3. Protein^+^ Drink Effect within the No-Muscle Insulin Resistance Subgroup

Table 4 presents the intervention effect of the no-MIR subgroup. A significantly greater change over time was seen for the protein^+^ drink on calcidiol (*p* = 0.001), HbA1c (*p* = 0.024), DI (*p* = 0.045), and 10-RM leg press (*p* = 0.026). Post-hoc evaluation showed increased calcidiol levels in the protein^+^ group (+19.1 nmol/L, *p* = 0.002) and improvements in both intervention groups, but stronger in the control group: HbA1c (control: −7.69 mmol/mol, *p* < 0.001; test: −2.92 mmol/mol, *p* = 0.040), was reduced after 13 weeks of intervention, while DI (control: +0.12, *p* = 0.024; test: −0.01, NS) and 10-RM leg press (control: +63.1, *p* < 0.001; test: +36.9, *p* < 0.001) were increased after 13 weeks of intervention. 

**Figure 1:**
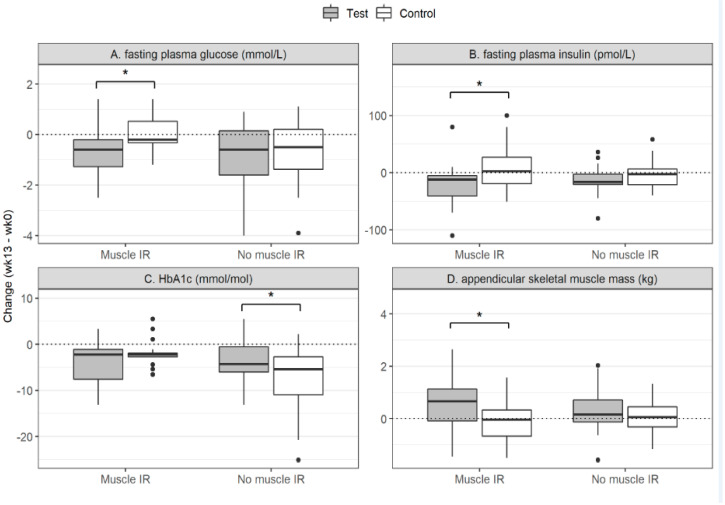
The changes in glycemic and physiological variables due to the intervention. HbA1c: glycated hemoglobin A1c; IR: insulin resistance; MIR: muscle insulin resistance. The changes in the fasting plasma glucose (**A**), fasting plasma insulin (**B**), HbA1c (**C**) and in appendicular skeletal muscle mass (**D**) are presented for the MIR group (left) and the no-MIR group (right) in each plot. The filled boxed (grey) represent the group supplemented with the protein drink; the non-filled boxed (white) represent the group provided with the control drink. Statistics was done within the MIR and no-MIR group and not between the groups. The groups are shown in the same plot for comparison. The box plots show quartile 1-quartile 3 of the data with the line representing the median. * *p* < 0.05.

**Figure 2:**
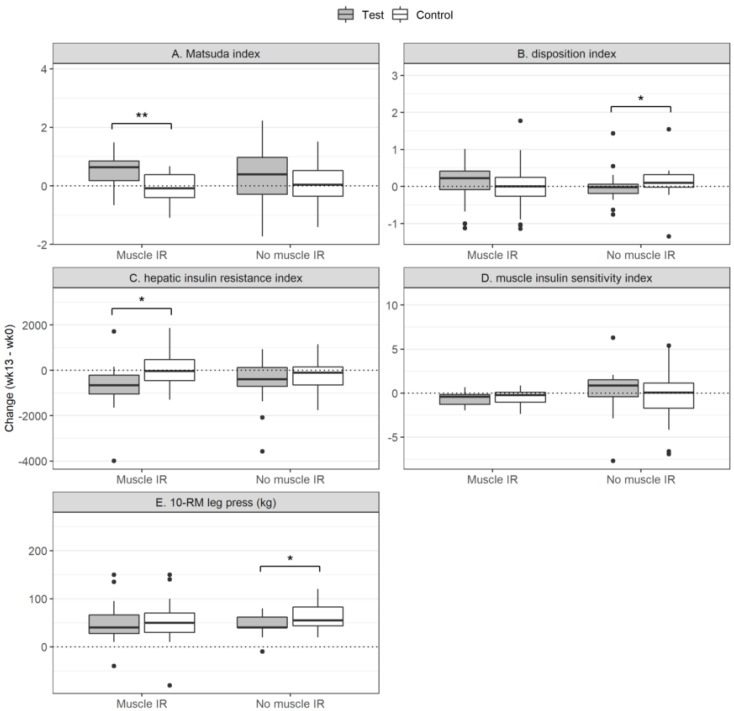
Insulin resistance indices and the 10-Repetition Max (10-RM) test. The changes in the Matsuda index (**A**), the Disposition Index (**B**), the Hepatic Insulin Resistance Index (**C**), the Muscle Insulin Sensitivity Index (**D**) and the 10-RM leg press (**E**) are presented for the MIR group (left) and the no-MIR group (right) in each plot. The filled boxed (grey) represent the group supplemented with the protein drink; the non-filled boxed (white) represent the group provided with the control drink. Statistics was done within the MIR and no-MIR group and not between the groups. The groups are shown in the same plot for comparison. The box plots show quartile 1-quartile 3 of the data with the line representing the median. * *p* < 0.05; ** *p* < 0.01.

In Figure 1 the changes of the glycemic and physiological parameters are presented for the test and the control group of the MIR and the no-MIR group. In Figure 2 the changes in the IR indices and 10-RM are shown for the test and control group for the MIR and the no-MIR group. 

We were also interested to evaluate the effect of the lifestyle intervention itself within the two subgroups. The effect of diet and exercise for both subgroups is presented in Table 3 and Table 4 as ‘Time effect’. In the MIR subgroup (Table 3), a significant time effect was found for body weight (−2.7 kg, *p* < 0.001), waist circumference (−3.0 cm, *p* < 0.001), BMI (−0.9 kg/m^2^, *p* < 0.001), systolic blood pressure (−8 mmHg, *p* = 0.05), HbA1c (−3.0 mmol/mol, *p* = 0.04), fat mass (−2.8 kg, *p* < 0.001), VAT (−22 cm^2^, *p* < 0.01), 10-RM leg press (+50 kg, *p* < 0.001), knee extension power (+20 Watt, *p* = 0.01), and VO_2_peak (+0.19 L/min, *p* < 0.001). All these parameters improved after 13 weeks of combined lifestyle intervention within the MIR subgroup, independent of the study drink (Table 3). Plasma cholesterol and triglyceride concentrations, Disposition Index (DI) and MISI did not change over time. 

In the no-MIR subgroup, presented in Table 4, a significant time effect was found for body weight (−2.7 kg, *p* < 0.001), waist circumference (−3.6 cm, *p* < 0.001), BMI (−0.9 kg/m^2^, *p* < 0.001), diastolic blood pressure (−5 mmHg, *p* = 0.004), fasting plasma glucose (−0.9 mmHg, *p* = 0.035), 2hr plasma glucose (−1.3 mmol/L, *p* = 0.001), HDL cholesterol (+0.1 mmol/L, *p* = 0.048), fat mass (−2.6 kg, *p* < 0.001), VAT (−23 cm^2^, *p* < 0.001), and knee extension power (+13 Watt, *p* = 0.01). All these parameters improved in both the control and test group after 13 weeks of combined lifestyle intervention, independent of the study drink. Systolic blood pressure, FPI, total and LDL cholesterol, triglycerides, ASMM, Matsuda, HIRI, MISI, and Adipo-IR did not change significantly over time in the no-MIR group.

**Figure 3:**
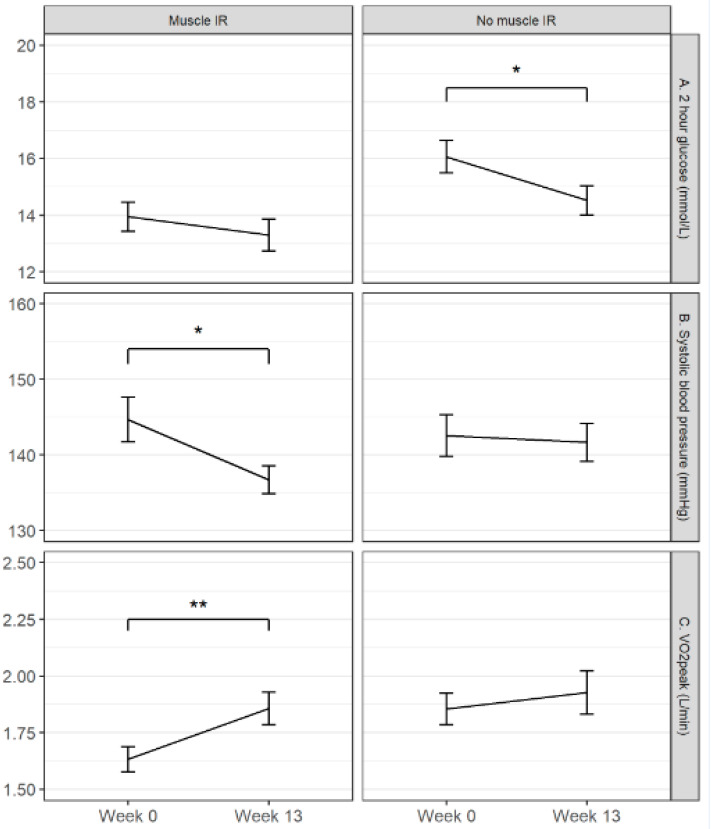
The lifestyle specific effects for the MIR and no-MIR group. The lines represent the changes in the 2 h glucose level (**A**), Systolic Blood Pressure (**B**) and VO_2_peak (**C**) due to the intervention from baseline to week 13 in the MIR (left side) and the no-MIR (right side) of the figure. The 2-h glucose level decreased significantly only in the no-MIR group; systolic blood pressure decreased only significantly in the MIR subgroup; and VO_2_peak increased only significantly in the MIR group. These changes were independent of treatment and due to time (lifestyle intervention effect). * *p* < 0.05; ** *p* < 0.01.

So besides similar effects of lifestyle on both subgroups, the systolic Blood Pressure (BP), HbA1c, 10-RM leg press and VO_2_peak were specific lifestyle effects found for the MIR subgroup. In the no-MIR group, the HbA1c and 10-RM leg press lifestyle effectiveness could not be determined due to the interaction effect with the protein^+^ drink. A specific lifestyle effect only seen in the no-MIR subgroup was the 2hr plasma glucose level, which was reduced. The lifestyle specific effects for the MIR and no-MIR subgroups on 2-h glucose, systolic blood pressure and VO_2_peak are shown in Figure 3.

## 4. Discussion

The present study indicates that obese, older type 2 diabetes patients with muscle IR who use the protein drink enriched with leucine and vitamin D during a combined lifestyle intervention, show benefits on appendicular skeletal muscle mass, fasting plasma glucose, fasting plasma insulin, systemic insulin sensitivity and adipose tissue insulin resistance.

Earlier publications showed specific effects of interventions on type 2 diabetes subgroups [17,28]. We hypothesized that especially subjects with muscle insulin resistance may benefit from additional whey protein intake as provided by the protein drink, since exercise specifically improves insulin sensitivity of the muscle [29,30] and the protein^+^ drink may increase the muscle glucose uptake capacity via muscle mass increase. Also of vitamin D beneficial effects for muscle growth and function have been reported [31]. The enriched protein supplement clearly resulted in increased Calcidiol levels, as a marker of vitamin D, after 13 weeks of intake in both subgroups. The role of vitamin D on insulin resistance has been reported, but recent reviews stressed that the relation is still not clear [32] and that there is no evidence that vitamin D supplementation has a beneficial effect on peripheral insulin sensitivity in people with or at risk of insulin resistance [33], as is stressed by our results as well. Mechanisms to elucidate these test product-driven beneficial effects specifically in the MIR subgroup, may therefore be related to the protein^+^ intake. The increased appendicular muscle mass of the MIR subgroup that consumed the protein^+^ drink suggests that the anabolic process is stimulated in this subgroup. Insulin not only stimulates glucose uptake in muscle, it is also an anabolic hormone, stimulating uptake of amino acids for muscle growth [34,35,36,37]. The MIR subgroup supplemented with the protein^+^ drink (whey protein) is becoming more insulin sensitive (tendency *p* = 0.08, see Table 3). The availability of the proper amino acids, as well as the increased training stimuli and the tendency for improved insulin sensitivity, are all factors favoring muscle growth [35]. The muscle IR of the no-MIR group did not improve at all and was already in the healthy range what may explain that no significant muscle growth was seen in the no-MIR group using protein^+^ drink.

Hepatic insulin sensitivity also improved significantly upon consumption of the protein^+^ drink in the MIR subgroup, suggesting that the relative increase in dietary protein and maybe the relative decrease in carbohydrate intake, also plays a role in hepatic insulin sensitivity. The lower relative carbohydrate content of subjects’ diets in the protein^+^ group as compared to control (daily about 20 g of carbohydrates less in the protein^+^ group) may have contributed to the reduction in fasting plasma glucose and insulin, adipose tissue insulin resistance, and the increase in systemic insulin sensitivity [38,39]. While we observed these beneficial effects of the protein^+^ drink compared to the control drink in the MIR subgroup, we did not observe them in the no-MIR subgroup, suggesting that these protein^+^ drink effects may be MIR subgroup-specific. Whether the results found are a consequence of increased protein or decreased carbohydrate intake needs further investigation.

In all groups the HbA1c level decreased during the intervention and a clear decline in the HbA1C level in the no-MIR subgroup on the protein^+^ and the control product was observed, whereas the decline in the no-MIR subgroup on control product was more pronounced and significantly different from the protein^+^ group. Also, the Disposition Index was significantly different between protein^+^ and control of the no-MIR group, where Disposition Index changed in the beneficial direction for control. Taken together, the clear beneficial effects of the protein^+^ drink in the MIR group were not observed in the no-MIR group. We cannot explain these findings and, therefore, this subgroup analysis needs to be confirmed and conducted in future research focusing on efficacy of protein supplementation in obese older type 2 diabetes subjects. 

In the present study, beneficial effects of the combined lifestyle intervention itself were seen in both the MIR and the no-MIR subgroup, with clinically relevant improvements in body weight, waist circumference, BMI, fat mass, visceral fat, hyperglycemia and knee extension power. Such effects are commonly reported for combined lifestyle interventions [40,41], with the exercise component being important to sustain health improvements obtained [42]. Remarkably, systolic blood pressure (SBP) improved (from 144 to 137 mm Hg, *p* = 0.02) due to the combined lifestyle intervention in the MIR subgroup only, independent of treatment. It is known that exercise training reduces SBP [43], probably due to improved endothelial function [44]. Our observation that this was only present in the MIR and not in the no-MIR subgroup suggests that MIR subgroup-specific effects of the lifestyle intervention occurred, as was also observed for the effects on VO_2_peak. A possible underlying explanation could be improved vasodilation and improved muscle perfusion in MIR subgroup only [45,46]. Due to the combined lifestyle intervention muscle blood flow may increase and result in the increased VO_2_peak. An increased blood flow by improved vasodilation could also explain the health benefits observed in the MIR subgroup when supplemented with a protein^+^ drink, like the increased appendicular skeletal muscle mass.

The muscle insulin resistance we focused on in the present study is suggested to be related to mitochondrial dysfunction [47,48]. It is known that exercise can improve mitochondrial oxidative capacity and has the potential to improve insulin sensitivity of the muscle [49]. In the MIR subgroup the combined lifestyle intervention had the tendency to improve muscle insulin sensitivity independent of the study drink (*p* = 0.08). It is suggested that disturbed muscle fat oxidative capacity is important for the development of muscle insulin resistance. This impaired oxidative capacity of the mitochondria in the muscle results in accumulation of intermediates of lipolysis, which could, in turn, interfere with insulin signaling and lead to inhibition of insulin-mediated glucose uptake [28,50]. Multiple physiological aspects of fat metabolism in muscle like ectopic fat storage, skeletal muscle fatty acid handling, and lipotoxicity may play a role in muscle insulin resistance. Scientific evidence is available, showing that disturbed fat metabolism in muscle is related to insulin resistance [50,51,52,53,54,55]. Whether this is the cause or consequence of diabetes development is still under debate.

In the present retrospective subgroup analysis we had a balanced distribution of MIR and no-MIR subjects, with protein^+^ and control (20–22 subjects), enabling us to perform the statistical subgroup analyses. The subgroups appeared to be less balanced when examining possible confounding variables like type 2 diabetes status (pre-diabetic or type 2 diabetes) and duration of type 2 diabetes (see Table 2). It was hypothesized that the MIR subgroup would be representative of type 2 diabetes patients with the most severe diabetes status. However, more pre-diabetics were present in the MIR (11 subjects) versus the no-MIR group (2 subjects). This could also partially explain the shorter duration of diabetes in the MIR subgroup (around 45 months for the MIR group and around 100 months in no-MIR group). This may have confounded the MIR versus no-MIR subtype intervention effects, which may relate to the progression of the disease rather than to the subtype itself. Patients with a shorter duration of type 2 diabetes may be more responsive to the combined lifestyle intervention and protein^+^ drink, which has been observed for type 2 diabetes remission with lifestyle [56]. In our study, the more severe type 2 diabetes patients were more often characterized as no-MIR. At this stage, it is unclear how type 2 diabetes disease progression is related to muscle insulin resistance and this requires further research.

The diabetes subgroups shown in the present study, were based upon the calculation of the muscle insulin sensitivity index. The use of organ-specific IR indices for subgroup analysis has been done by others as well [52,53,54,57]. They stress, like we do, that this in-depth phenotyping of subjects provides more insight into disturbed underlying disease mechanisms. It is furthermore suggested that the use of organ-specific IR indices may be interesting and useful in personalization of type 2 diabetes treatment, and treatment effect evaluation [52,53,54]. 

In our study, the following strengths and limitations are present. A strength of the present study is the use of data of a well-performed randomized-controlled, double-blind trial, the PROBE study [14]. The number of subjects present in our subgroups was nicely balanced and enabled the within-group comparison. Limitations of the study can also be identified. The small group sizes and relatively large standard deviations for some parameters may have limited the ability to obtain statistical significance. Multiple testing correction was not applied because of the exploratory nature of this work. The subgroup analysis performed was done retrospectively, thus could not be sufficiently powered and balanced by rules of controlled trials and a risk of type II errors with the statistical analysis is present. Follow-up studies are needed to confirm the promising effect of the enriched protein supplement in combination with lifestyle intervention for subgroups of type 2 diabetic patients. 

## 5. Conclusions

The results of our retrospective study indicate that especially obese, older type 2 diabetes patients with muscle IR may benefit from the use of a vitamin D and leucine enriched whey protein drink during combined lifestyle intervention, by showing improved systemic insulin sensitivity, adipose tissue insulin resistance, and appendicular muscle mass. The combined lifestyle intervention itself was also more effective in the MIR subgroup. Awaiting confirmation by future controlled trials, this study indicates subgroup-specific lifestyle intervention responses and reiterates the potential for a personalized lifestyle and diet approach to support patients with type 2 diabetes.

## Figures and Tables

**Table 1:** Indices used to define the diabetic phenotype of a subject.

Index	Formula	Indicator of
Matsuda Index [19]	10000/√(fG × fI)(mG x mI)	Systemic insulin sensitivity
Disposition Index (DI) [20]	[AUC_30min insulin_/AUC_30min glucose_] × Matsuda	β-cell function
Hepatic Insulin Resistance Index (HIRI) [18]	fG × fI	Hepatic insulin resistance
Muscle Insulin Sensitivity Index (MISI) [18]	(∆G/∆t)/mI	Muscle insulin resistance
Adipose Tissue Insulin Resistance Index (Adipo-IR) [21]	Fasting FFA × fI	Adipose tissue insulin resistance

Abbreviations used: fG = fasting plasma glucose; fI = fasting plasma insulin; mG = mean plasma glucose; mI = mean plasma insulin; AUC = area under the curve; ∆G = delta glucose; ∆t = delta time; FFA = free fatty acids. Units used: for glucose mg/dL; for insulin U/L; for FFA mmol/L.

**Table 2:** Descriptive characteristics of the subjects.

	MIR ^+^		no-MIR	
	Test	Control	Test	Control
Number	20	22	20	20
Gender (male/female)	12/8	11/11	14/6	16/4
Age (years) (mean ± SD ^++^)	67.3 ± 5.4	67.1 ± 5.7	68.3 ± 5.6	63.6 ± 5.9
T2D ^@^ duration (months); median (IQR ^#^)	56 (40–100)	32 (15–51)	100 (59–162)	110 (69–132)
Diabetes status (T2DM/pre-diabetes) *	13/7	18/4	19/1	19/1
Use of Metformin (yes/no)	11/9	16/6	19/1	19/1
Use of SU derivatives (yes/no)	7/13	6/16	5/15	9/11

**^+^** MIR: Muscle-Insulin Resistance; **^++^** SD: Standard Deviation; ^@^ T2D: type 2 diabetes; ^#^ IQR: interquartile range of q1–q3. * Based upon diabetes medication at screening.

**Table 3:** Clinical characteristics in test and control group for subjects in muscle insulin resistance (MIR) subgroup.

	Test (*n* = 20)		Control (*n* = 22)		Time Effect	Interaction Effect (Time × Treatment)
	Week 0	Week 13	Week 0	Week 13	*p*-value	*p*-value
Weight (kg)	98.8 (14.8)	96.7 (14.4)	98.3 (16.2)	95.7 (16.2)	**<0.001**	0.67
Waist circumference (cm)	115.8 (8.0)	113.3 (8.6)	117.2 (10.7)	112.4 (8.9)	**<0.001**	0.35
BMI (kg/m^2^)	34.0 (4.4)	33.2 (4.2)	33.6 (4.6)	32.8 (4.9)	**<0.001**	0.71
Systolic blood pressure (mm Hg)	144 (20)	136 (13)	145 (18)	138 (11)	**0.02**	0.87
Diastolic blood pressure (mm Hg)	86 (10)	80 (9)	89 (8)	85 (7)	0.05	0.62
Fasting plasma glucose (mmol/L)	7.8 (1.3)	7.1 (1.1)	7.4 (1.1)	7.3 (1.2)		**0.02 ^^^**
2hr plasma glucose (mmol/L)	14.0 (3.4)	13.5 (3.1)	13.9 (3.3)	13.1 (4.1)	0.10	0.64
HbA1c (mmol/mol)	47.84 (5.77)	44.60 (4.78)	46.72 (6.43)	44.22 (4.87)	**0.04**	0.24
Fasting plasma insulin (pmol/L)	135.9 (78.1)	115.1 (55.9)	116.6 (36.9)	126.3 (57.8)		**0.03 ^^^**
Total cholesterol (mmol/L)	4.1 (1.0)	4.1 (1.1)	4.2 (0.8)	4.0 (0.7)	0.24	0.56
HDL cholesterol (mmol/L)	1.14 (0.31)	1.21 (0.27)	1.15 (0.23)	1.19 (0.18)	0.11	0.54
LDL cholesterol (mmol/L)	2.2 (0.8)	2.1 (0.8)	2.3 (0.6)	2.2 (0.6)	0.15	0.72
Triglycerides (mmol/L)	1.5 (0.4)	1.4 (0.3)	1.5 (0.5)	1.5 (0.4)	0.66	0.32
Calcidiol (nmol/L)	56.1 (22.1)	73.5 (13.8)	58.1 (17.3)	55.2 (21.1)		**<0.001 ^^^**
Leucine	0.20 (0.04)	0.20 (0.03)	0.19 (0.04)	0.19 (0.04)	0.19	0.74
Fat mass (kg)	36.0 (10.0)	32.7 (9.0)	35.3 (8.0)	32.5 (8.5)	**<0.001**	0.28
VAT (cm^2^)	197 (52)	172 (41)	189 (51)	166 (46)	**<0.01**	0.61
ASMM (kg)	25.4 (5.1)	26.3 (5.3)	25.5 (5.5)	25.5 (5.6)		**0.04 ^^^**
Matsuda index	1.63 (0.59)	2.10 (0.81)	1.82 (0.69)	1.71 (0.83)		**<0.01 ^^^**
Disposition index	1.23 (1.43)	1.31 (1.44)	1.44 (2.28)	1.43 (2.12)	0.60	0.17
Hepatic insulin resistance index	2868.5 (2113.5)	2168.8 (1324.8)	2237.7 (743.3)	2421.12 (1193.1)		**0.02 ^^^**
Muscle insulin sensitivity index	−0.41 (0.34)	−1.01 (0.80)	−0.39 (0.32)	−0.83 (0.79)	0.08	0.47
Adipose tissue insulin resistance index	92.90 (59.20)	69.13 (39.99)	75.96 (30.15)	77.17 (39.70)		**0.04 ^^^**
10-RM leg press (kg)	159 (78)	213 (73)	119 (45)	161 (66)	**<0.001**	0.73
Knee extension power (Watt)	186 (84)	210 (69)	172 (50)	184 (56)	**0.01**	0.90
VO_2_peak (L/min)	1.68 (0.37)	1.89 (0.39)	1.58 (0.33)	1.83 (0.51)	**<0.001**	0.45

Data given are as mean and standard deviation (SD). HbA1c: glycated hemoglobin A1c; HDL: High-Density Lipoprotein; LDL: Low-Density Lipoprotein; BMI = body mass index; VAT: Visceral Adipose Tissue; ASMM = appendicular skeletal muscle mass. 10-RM = 10 repetitions max. ^^^ Significant effect (*p* < 0.05) in test group after post-hoc analysis.

**Table 4:** Clinical characteristics in test and control group for subjects in no-MIR subgroup.

	Test (*n* = 20)		Control (*n* = 20)		Time Effect	Interaction Effect (Time × Treatment)
	Week 0	Week 13	Week 0	Week 13	*p*-value	*p*-value
Weight (kg)	98.6 (15.5)	96.3 (14.4)	97.6 (12.4)	94.6 (12.0)	**<0.001**	0.42
Waist circumference (cm)	112.4 (12.4)	108.3 (11.4)	111.9 (9.0)	108.5 (9.6)	**<0.001**	0.54
BMI (kg/m^2^)	32.2 (4.3)	31.4 (3.8)	31.8 (2.5)	30.8 (2.6)	**<0.001**	0.47
Systolic blood pressure (mm Hg)	144 (19)	143 (17)	141 (16)	140 (14)	0.84	0.95
Diastolic blood pressure (mm Hg)	85 (9)	80 (6)	86 (9)	80 (9)	**0.004**	0.62
Fasting plasma glucose (mmol/L)	8.3 (1.5)	7.3 (1.3)	8.4 (1.9)	7.8 (1.9)	**0.035**	0.57
2hr plasma glucose (mmol/L)	15.2 (3.5)	14.2 (3.4)	17.0 (3.5)	14.8 (3.2)	**0.001**	0.23
HbA1c (mmol/mol)	49.79 (6.62)	46.86 (5.84)	53.63 (10.66)	46.68 (6.09)	.	**0.02 ^+^**
Fasting plasma insulin (pmol/L)	85.1 (39.1)	75.3 (29.8)	84.0 (30.1)	81.1 (24.3)	0.72	0.20
Total cholesterol (mmol/L)	3.9 (0.9)	3.8 (0.9)	3.8 (0.7)	3.8 (0.7)	0.59	0.38
HDL cholesterol (mmol/L)	1.17 (0.31)	1.18 (0.25)	1.07 (0.23)	1.14 (0.17)	**0.048**	0.60
LDL cholesterol (mmol/L)	2.0 (0.7)	1.9 (0.7)	2.1 (0.6)	2.0 (0.7)	0.29	0.83
Triglycerides (mmol/L)	1.6 (0.6)	1.4 (0.7)	1.4 (0.7)	1.3 (0.5)	0.50	0.30
Calcidiol (nmol/L)	67.4 (29.3)	89.3 (17.6)	59.8 (17.3)	55.5 (18.3)		**<0.01 ^^^**
Leucine	0.19 (0.02)	0.19 (0.03)	0.19 (0.03)	0.19 (0.03)	0.42	0.70
Fat mass (kg)	32.5 (9.9)	29.7 (9.0)	30.3 (6.3)	27.9 (6.4)	**<0.001**	0.51
VAT (cm^2^)	181 (73)	156 (64)	181 (49)	158 (50)	**<0.001**	0.36
ASMM (kg)	26.8 (5.0)	27.6 (5.3)	27.9 (4.4)	27.9 (4.4)	0.66	0.29
Matsuda index	2.68 (1.23)	2.93 (1.48)	2.62 (0.89)	2.70 (0.66)	0.66	0.35
Disposition index	0.88 (0.55)	0.84 (0.66)	0.71 (0.67)	0.83 (0.48)	.	**0.045 ^#^**
Hepatic insulin resistance index	2012.9 (1216.5)	1541.5 (752.4)	1912.0 (829.6)	1632.5 (558.3)	0.17	0.26
Muscle insulin sensitivity index	−3.25 (2.72)	−2.99 (3.07)	−3.25 (1.72)	−3.83 (2.64)	0.91	0.37
Adipose tissue insulin resistance index	62.71 (46.29)	44.52 (21.56)	53.09 (20.65)	48.66 (21.61)	0.21	0.58
10-RM leg press (kg)	124 (36)	178 (66)	148 (67)	220 (79)		0.03 *
Knee extension power (Watt)	184 (48)	200 (47)	203 (72)	229 (74)	**0.01**	0.98
VO_2_peak (L/min)	1.68 (0.50)	1.76 (0.63)	2.03 (0.30)	2.08 (0.45)	0.60	0.43

Data given are as mean and standard deviation (SD). HbA1c: glycated hemoglobin A1c; HDL: High-Density Lipoprotein; LDL: Low-Density Lipoprotein; BMI = body mass index; VAT: Visceral Adipose Tissue; ASMM = appendicular skeletal muscle mass. 10-RM = 10 repetitions max. ^^^ Significant effect (*p* < 0.05) in test group after post-hoc analysis, ^+^ Significant effect (*p* < 0.05) in control group after post-hoc analysis, ^#^ no significant (*p* > 0.05) post-hoc effects, * significant effect (*p* < 0.001) in both test and control group after post hoc analysis.

## Appendix A

**Table A1:** Composition of the Study Products, per Serving of 150 mL.

Component	Test Product Protein^+^	Control Product
Energy (kcal)	150	150
Protein (g)		
Total (g)	20.7	-
Leucine, total ^a^ (g)	2.8	-
EAA, total ^a^ (g)	10.6	-
Carbohydrates		
Total (g)	9.4	24.5
Sugars ^b^ (g)	4.2	15.6
Fat		
Total (g)	3.0	5.8
Saturated (g)	0.8	3.2
Mono-unsaturated (g)	1.7	2.1
Poly-unsaturated (g)	0.6	0.6
Fiber (g)	1.25	-
Vitamin D_3_^c^ (µg)	20	-
Calcium ^c^ (mg)	500	0.7

EAA, essential amino acids (Leu, Ile, Val, Phe, Met, His, Trp, Thr, and Lys); BCAA: branched-chain amino acids (Leu, Ile, and Val). ^a^ Provided by protein and free BCAA. ^b^ Consisting of lactose and fructose. ^c^ The test product also contained the following micronutrients: phosphorus (250 mg), magnesium (37 mg), iron (2.4 mg), zinc (2.2 mg), copper (270 mg), manganese (0.50 mg), fluoride (0.15 mg), molybdenum (15 µg), selenium (15 µg), chromium (7.5 µg), iodine (20 µg), vitamin A (152 µg retinol equivalents), vitamin E (7.5 mg α-tocopherol equivalents), vitamin K (12 µg), vitamin B-1 (0.23 mg), vitamin B-2 (0.25 mg), niacin (8.8 mg niacin equivalents), pantothenic acid (0.81 mg), vitamin B-6 (750 µg), folic acid (200 µg), vitamin B-12 (3.0 µg), biotin (6.1 µg), vitamin C (32 mg), carotenoids (300 µg), and choline (55 mg). Protein+, an enriched protein drink.

## References

[1] Lim E.L., Hollingsworth K.G., Aribisala B.S., Chen M.J., Mathers J.C., Taylor R. (2011). Reversal of Type 2 Diabetes: Normalisation of Beta Cell Function in Association with Decreased Pancreas and Liver Triacylglycerol. Diabetologia.

[2] Steven S., Hollingsworth K.G., Small P.K., Woodcock S.A., Pucci A., Aribisala B., Al-Mrabeh A., Daly A.K., Batterham R.L., Taylor R. (2016). Weight Loss Decreases Excess Pancreatic Triacylglycerol Specifically in Type 2 Diabetes. Diabetes Care.

[3] Steven S., Hollingsworth K.G., Al-Mrabeh A., Avery L., Aribisala B., Caslake M., Taylor R. (2016). Very Low-Calorie Diet and 6 Months of Weight Stability in Type 2 Diabetes: Pathophysiological Changes in Responders and Nonresponders. Diabetes Care.

[4] Lean M.E., Leslie W.S., Barnes A.C., Brosnahan N., Thom G., McCombie L., Peters C., Zhyzhneuskaya S., Al-Mrabeh A., Hollingsworth K.G. (2018). Primary Care-Led Weight Management for Remission of Type 2 Diabetes (DiRECT): An Open-Label, Cluster-Randomised Trial. Lancet.

[5] Wycherley T.P., Buckley J.D., Noakes M., Clifton P.M., Brinkworth G.D. (2013). Comparison of the Effects of Weight Loss from a High-Protein versus Standard-Protein Energy-Restricted Diet on Strength and Aerobic Capacity in Overweight and Obese Men. Eur. J. Nutr..

[6] Villareal D.T., Chode S., Parimi N., Sinacore D.R., Hilton T., Armamento-Villareal R., Napoli N., Qualls C., Shah K. (2011). Weight Loss, Exercise, or Both and Physical Function in Obese Older Adults. N. Engl. J. Med..

[7] Rosenberg I.H. (1997). Sarcopenia: Origins and Clinical Relevance. J. Nutr..

[8] Seok W.P., Goodpaster B.H., Jung S.L., Kuller L.H., Boudreau R., De Rekeneire N., Harris T.B., Kritchevsky S., Tylavsky F.A., Nevitt M. (2009). Excessive Loss of Skeletal Muscle Mass in Older Adults with Type 2 Diabetes. Diabetes Care.

[9] Thiebaud D., Jacot E., DeFronzo R.A., Maeder E., Jequier E., Felber J.-P.P. (1982). The Effect of Graded Doses of Insulin on Total Glucose Uptake, Glucose Oxidation, and Glucose Storage in Man. Diabetes.

[10] Willey K.A., Fiatarone Singh M.A. (2003). Battling Insulin Resistance in Elderly Obese People with Type 2 Diabetes: Bring on the Heavy Weights. Diabetes Care.

[11] Trouwborst I., Verreijen A., Memelink R., Massanet P., Boirie Y., Weijs P., Tieland M. (2018). Exercise and Nutrition Strategies to Counteract Sarcopenic Obesity. Nutrients.

[12] Frimel T.N., Sinacore D.R., Villareal D.T. (2008). Exercise Attenuates the Weight-Loss-Induced Reduction in Muscle Mass in Frail Obese Older Adults. Med. Sci. Sports Exerc..

[13] Verreijen A.M., Verlaan S., Engberink M.F., Swinkels S., de Vogel-van den Bosch J., Weijs P.J.M. (2015). A High Whey Protein-, Leucine-, and Vitamin D-Enriched Supplement Preserves Muscle Mass during Intentional Weight Loss in Obese Older Adults: A Double-Blind Randomized Controlled Trial. Am. J. Clin. Nutr..

[14] Memelink R., Pasman W., Bongers A., Tump A., van Ginkel A., Tromp W., Wopereis S., Verlaan S., de Vogel-van den Bosch J., Weijs P. (2018). Effect of a Whey Protein Drink Enriched with Leucine and Vitamin D on Lean Mass and Glycemic Control during a Lifestyle Intervention in Obese Older Adults with (Pre-)Diabetes Type 2: A Double-Blind RCT. Clin. Nutr..

[15] Van Ommen B., van der Greef J., Ordovas J.M., Daniel H. (2014). Phenotypic Flexibility as Key Factor in the Human Nutrition and Health Relationship. Genes Nutr..

[16] DeFronzo R.A. (2010). Insulin Resistance, Lipotoxicity, Type 2 Diabetes and Atherosclerosis: The Missing Links. The Claude Bernard Lecture 2009. Diabetologia.

[17] Blanco-Rojo R., Alcala-Diaz J.F., Wopereis S., Perez-Martinez P., Quintana-Navarro G.M., Marin C., Ordovas J.M., van Ommen B., Perez-Jimenez F., Delgado-Lista J. (2016). The Insulin Resistance Phenotype (Muscle or Liver) Interacts with the Type of Diet to Determine Changes in Disposition Index after 2 Years of Intervention: The CORDIOPREV-DIAB Randomised Clinical Trial. Diabetologia.

[18] Abdul-Ghani M.A., Matsuda M., Balas B., DeFronzo R.A. (2007). Muscle and Liver Insulin Resistance Indexes Derived from the Oral Glucose Tolerance Test. Diabetes Care.

[19] Matsuda M., Defronzo R.A. (1999). Insulin Sensitivity Indices Obtained From Comparison with the Euglycemic Insulin Clamp. Diabetes Care.

[20] Kahn S.E., Prigeon R.L., Mcculloch D.K., Boyko E.J., Bergman R.N., Schwartz M.W., Neifing J.L., Ward W.K., Beard J.C., Palmer J.P. (1993). Quantification of the Relationship Between Insulin Sensitivity and P-Cell Function in Human Subjects Evidence for a Hyperbolic Function with a Regulated Feedback Loop Control System Such That for Any Difference in S, a Proportionate Reciprocal Difference. Diabetes.

[21] Ter Horst K.W., Van Galen K.A., Gilijamse P.W., Hartstra A.V., De Groot P.F., Van Der Valk F.M., Ackermans M.T., Nieuwdorp M., Romijn J.A., Serlie M.J. (2017). Methods for Quantifying Adipose Tissue Insulin Resistance in Overweight/Obese Humans. Int. J. Obes..

[22] Larsen T.M., Dalskov S., Van Baak M., Jebb S., Kafatos A., Pfeiffer A., Martinez J.A., Handjieva-Darlenska T., Kunešová M., Holst C. (2010). The Diet, Obesity and Genes (Diogenes) Dietary Study in Eight European Countries—A Comprehensive Design for Long-Term Intervention. Obes. Rev..

[23] Delgado-Lista J., Perez-Martinez P., Garcia-Rios A., Alcala-Diaz J.F., Perez-Caballero A.I., Gomez-Delgado F., Fuentes F., Quintana-Navarro G., Lopez-Segura F., Ortiz-Morales A.M. (2016). CORonary Diet Intervention with Olive Oil and Cardiovascular PREVention Study (the CORDIOPREV Study): Rationale, Methods, and Baseline Characteristics A Clinical Trial Comparing the Efficacy of a Mediterranean Diet Rich in Olive Oil versus a Low-Fat Diet. Am. Heart J..

[24] Wopereis S., Stroeve J.H.M., Stafleu A., Bakker G.C.M., Burggraaf J., van Erk M.J., Pellis L., Boessen R., Kardinaal A.A.F., van Ommen B. (2017). Multi-Parameter Comparison of a Standardized Mixed Meal Tolerance Test in Healthy and Type 2 Diabetic Subjects: The PhenFlex Challenge. Genes Nutr..

[25] Van Den Broek T.J., Bakker G.C.M.M., Rubingh C.M., Bijlsma S., Stroeve J.H.M.M., Van Ommen B., Van Erk M.J., Wopereis S. (2017). Ranges of Phenotypic Flexibility in Healthy Subjects. Genes Nutr..

[26] Neeter C., Gustavsson A., Thomeé P., Augustsson J., Thomeé R., Karlsson J. (2006). Development of a Strength Test Battery for Evaluating Leg Muscle Power after Anterior Cruciate Ligament Injury and Reconstruction. Knee Surg. Sports Traumatol. Arthrosc..

[27] Praet S.F.E., Van Loon L.J.C. (2007). Optimizing the Therapeutic Benefits of Exercise in Type 2 Diabetes. J. Appl. Physiol..

[28] Blaak E.E. (2005). Metabolic Fluxes in Skeletal Muscle in Relation to Obesity and Insulin Resistance. Best Pract. Res. Clin. Endocrinol. Metab..

[29] Goodpaster B.H., Sparks L.M. (2017). Metabolic Flexibility in Health and Disease. Cell Metab..

[30] Cartee G.D., Hepple R.T., Bamman M.M., Zierath J.R. (2016). Exercise Promotes Healthy Aging of Skeletal Muscle. Cell Metab..

[31] Garcia M., Seelaender M., Sotiropoulos A., Coletti D., Lancha A.H. (2019). Vitamin D, Muscle Recovery, Sarcopenia, Cachexia, and Muscle Atrophy. Nutrition.

[32] Sacerdote A., Dave P., Lokshin V., Bahtiyar G. (2019). Type 2 Diabetes Mellitus, Insulin Resistance, and Vitamin D. Curr. Diabetes Rep..

[33] Pramono A., Jocken J.W.E., Blaak E.E., van Baak M.A. (2020). The Effect of Vitamin D Supplementation on Insulin Sensitivity: A Systematic Review and Meta-Analysis. Diabetes Care.

[34] Rhoads R.P., Baumgard L.H., El-Kadi S.W., Zhao L.D. (2016). Roles for Insulin-Supported Skeletal Muscle Growth. J. Anim. Sci..

[35] Stokes T., Hector A.J., Morton R.W., McGlory C., Phillips S.M. (2018). Recent Perspectives Regarding the Role of Dietary Protein for the Promotion of Muscle Hypertrophy with Resistance Exercise Training. Nutrients.

[36] Abdulla H., Smith K., Atherton P.J., Idris I. (2016). Role of Insulin in the Regulation of Human Skeletal Muscle Protein Synthesis and Breakdown: A Systematic Review and Meta-Analysis. Diabetologia.

[37] James H.A., O’Neill B.T., Nair K.S. (2017). Insulin Regulation of Proteostasis and Clinical Implications. Cell Metab..

[38] Krebs J., Hall R., Parry Strong A. (2016). Importance of Low Carbohydrate Diets in Diabetes Management. Nutr. Diet. Suppl..

[39] Van Zuuren E.J., Fedorowicz Z., Kuijpers T., Pijl H. (2018). Effects of Low-Carbohydrate-Compared with Low-Fat-Diet Interventions on Metabolic Control in People with Type 2 Diabetes: A Systematic Review Including GRADE Assessments. Am. J. Clin. Nutr..

[40] Salas-Salvadó J., Díaz-López A., Ruiz-Canela M., Basora J., Fitó M., Corella D., Serra-Majem L., Wärnberg J., Romaguera D., Estruch R. (2019). Effect of a Lifestyle Intervention Program with Energy-Restricted Mediterranean Diet and Exercise on Weight Loss and Cardiovascular Risk Factors: One-Year Results of the PREDIMED-Plus Trial. Diabetes Care.

[41] Pot G.K., Ce Battjes-Fries M., Patijn O.N., Pijl H., Witkamp R.F., De Visser M., Van Der Zijl N., De Vries M., Voshol P.J., Bolk L. (2019). Nutrition and Lifestyle Intervention in Type 2 Diabetes: Pilot Study in the Netherlands Showing Improved Glucose Control and Reduction in Glucose Lowering Medication. BMJ Nutr. Prev. Health.

[42] Bouchonville M., Armamento-Villareal R., Shah K., Napoli N., Sinacore D.R., Qualls C., Villareal D.T. (2014). Weight Loss, Exercise or Both and Cardiometabolic Risk Factors in Obese Older Adults: Results of a Randomized Controlled Trial. Int. J. Obes..

[43] Cornelissen V.A., Smart N.A. (2013). Exercise Training for Blood Pressure: A Systematic Review and Meta-Analysis. Journal of the American Heart Association. J. Am. Heart Assoc..

[44] Vona M., Codeluppi G.M., Iannino T., Ferrari E., Bogousslavsky J., Von Segesser L.K. (2009). Effects of Different Types of Exercise Training Followed by Detraining on Endothelium-Dependent Dilation in Patients with Recent Myocardial Infarction. Circulation.

[45] Pinckard K., Baskin K.K., Stanford K.I. (2019). Effects of Exercise to Improve Cardiovascular Health. Front. Cardiovasc. Med..

[46] Keske M.A., Dwyer R.M., Russell R.D., Blackwood S.J., Brown A.A., Hu D., Premilovac D., Richards S.M., Rattigan S. (2017). Regulation of Microvascular Flow and Metabolism: An Overview. Clin. Exp. Pharm. Physiol..

[47] Di Meo S., Iossa S., Venditti P. (2017). Skeletal Muscle Insulin Resistance: Role of Mitochondria and Other ROS Sources. J. Endocrinol..

[48] Montgomery M.K., Turner N. (2015). Mitochondrial Dysfunction and Insulin Resistance: An Update. Endocr. Connect..

[49] Broskey N.T., Greggio C., Boss A., Boutant M., Dwyer A., Schlueter L., Hans D., Gremion G., Kreis R., Boesch C. (2014). Skeletal Muscle Mitochondria in the Elderly: Effects of Physical Fitness and Exercise Training. J. Clin. Endocrinol. Metab..

[50] Abdul-Ghani M.A., Defronzo R.A. (2010). Pathogenesis of Insulin Resistance in Skeletal Muscle. J. Biomed. Biotechnol..

[51] van der Kolk B.W., Goossens G.H., Jocken J.W., Blaak E.E. (2016). Altered Skeletal Muscle Fatty Acid Handling Is Associated with the Degree of Insulin Resistance in Overweight and Obese Humans. Diabetologia.

[52] van der Kolk B.W., Vogelzangs N., Jocken J.W.E., Valsesia A., Hankemeier T., Astrup A., Saris W.H.M., Arts I.C.W., van Greevenbroek M.M.J., Blaak E.E. (2019). Plasma Lipid Profiling of Tissue-Specific Insulin Resistance in Human Obesity. Int. J. Obes..

[53] Trouwborst I., Bowser S.M., Goossens G.H., Blaak E.E. (2018). Ectopic Fat Accumulation in Distinct Insulin Resistant Phenotypes; Targets for Personalized Nutritional Interventions. Front. Nutr..

[54] Blaak E.E. (2017). Characterisation of Fatty Acid Metabolism in Different Insulin-Resistant Phenotypes by Means of Stable Isotopes. Proc. Nutr. Soc..

[55] Meex R.C.R., Blaak E.E., van Loon L.J.C. (2019). Lipotoxicity Plays a Key Role in the Development of Both Insulin Resistance and Muscle Atrophy in Patients with Type 2 Diabetes. Obes. Rev..

[56] Taylor R., Al-Mrabeh A., Zhyzhneuskaya S., Peters C., Barnes A.C., Aribisala B.S., Hollingsworth K.G., Mathers J.C., Sattar N., Lean M.E.J. (2018). Erratum: Remission of Human Type 2 Diabetes Requires Decrease in Liver and Pancreas Fat Content but Is Dependent upon Capacity for β Cell Recovery. Cell Metab..

[57] Blaak E.E. (2020). Current metabolic perspective on malnutrition in obesity: Towards more subgroup-based nutritional approaches?. Proc. Nutr. Soc..

